# The impact of baseline laboratory tests on the management of new-onset hypertension in primary care: A pilot study

**DOI:** 10.1371/journal.pone.0324743

**Published:** 2025-05-29

**Authors:** Katia Kteich, Maria R. Karam, Marouan Zoghbi, Mabel Aoun

**Affiliations:** 1 Faculty of Medicine, Saint-Joseph University of Beirut, Beirut, Lebanon; 2 Fondation AUB Santé, Lorient, France; Tehran University of Medical Sciences, IRAN, ISLAMIC REPUBLIC OF

## Abstract

**Background:**

Hypertension is a key contributor to the global cardiovascular disease burden. In 2021, the World Health Organization (WHO) hypertension management guideline suggested baseline laboratory tests for patients with newly diagnosed hypertension but noted limited evidence in primary care contexts. This study evaluates the impact of baseline laboratory assessments on blood pressure control and comorbidities in newly diagnosed hypertensive patients.

**Methods:**

This is a multicenter retrospective study that included all patients with new-onset hypertension between January 2015 and January 2020, followed in three primary health care centers until 2022. Data collection included 8 items of paraclinical tests performed at the diagnosis of hypertension: serum sodium, potassium and creatinine, lipid panel, electrocardiogram, glucose, HbA1c and urine dipstick. Complete workup was defined as having the 8 items checked and partial workup included 1–7 items. Blood pressure was assessed at one year and the final visit, which was beyond one year, in the two workup groups.

**Results:**

Of 621 hypertensive patients, 107 with incident hypertension were analyzed (mean age: 54.8 ± 12.7 years; 58.9% women). A complete workup was done for 48 patients, partial for 52 and none for 7. Abnormalities detected included: 8.4% of patients with fasting blood glucose > 125 mg/dL, 7.5% with HbA1c > 6.5%, 1.9% with serum potassium < 3.5 mmoL/L, 54.2% with LDL Cholesterol > 100 mg/dL, 35.5% with serum creatinine > 0.8 mg/dL, and 7.5% with an estimated GFR < 60 mL/min/1.73 m2. Significant systolic blood pressure improvement was seen at 12 months in the complete workup group (129.9 ± 13.6 mmHg) vs. partial workup group (142.8 ± 18.9 mm Hg) (*P* = 0.003). Men and smokers were tested more often than women and non-smokers.

**Conclusion:**

Baseline laboratory tests in patients with newly diagnosed hypertension help unmask comorbidities such as chronic kidney disease, diabetes, and dyslipidemia. Our findings support the use of baseline laboratory testing in order to optimize blood pressure control and individual patient management.

## Introduction

According to the World Health Organization (WHO), cardiovascular diseases represent the leading cause of mortality worldwide. Three-quarters of deaths in low- and middle-income countries are caused by heart diseases [1]. Hypertension is one of the major cardiovascular risk factors. Over the past few decades, several guidelines and recommendations have been developed and incorporated into the management of hypertension, the latest being published by WHO in 2021 on the pharmacological treatment of hypertension, mainly targeting socio-economically disadvantaged settings [2].

Based on the WHO 2021 guideline, performing baseline paraclinical tests before initiating antihypertensive treatment is suggested, provided that it does not delay the initiation of treatment [2]. Tests suggested by the WHO guideline include serum electrolytes and creatinine, lipid panel, HbA1C or fasting glucose, urine dipstick, and electrocardiogram (ECG) [2]. Similarly, the European Society of Hypertension (ESH) and the American Heart Association/American College of Cardiology (AHA/ACC) recommend some laboratory tests for patients newly diagnosed with hypertension [3,4]. The ESH suggests additional tests such as hemoglobin/hematocrit, serum calcium, uric acid and urinary albumin to creatinine ratio (ACR), to be adapted based on clinical circumstances [3]. In contrast, the AHA/ACC suggests adding thyroid-stimulating hormone and complete blood count, while keeping uric acid and ACR as optional [4]. However, all these approaches lack strong evidence. To our knowledge, there are no comparative studies in the literature assessing the benefit of paraclinical tests for newly diagnosed hypertension.

The potential benefits of such assessment include the early diagnosis of secondary causes of hypertension, the detection of comorbidities (such as diabetes, dyslipidemia, or chronic kidney disease), the identification of target organ damage, and the possibility of stratifying patients into cardiovascular risk categories and prioritizing one class of medication over another. However, in an effort to reduce costs and/or avoid delaying treatment, all these mentioned benefits might be missed. It is uncertain whether baseline assessments, by detecting other comorbidities, contribute to better blood pressure control or a reduction in cardiovascular morbidity and mortality due to hypertension.

This study aims to identify the percentage of primary care patients with new-onset hypertension who underwent complete or partial laboratory testing, as well as the percentage of abnormal test results and the impact of testing on blood pressure control.

## Materials and methods

### Study participants and design

This is a multicenter, retrospective study including all patients with newly diagnosed hypertension in three large Lebanese primary healthcare centers (PHCCs) between 2015 and 2020, followed until 2022. The three PHCCS are located at Hôtel Dieu de France University Hospital, the Makhzoumi Foundation Center, and the Saint-Antoine Center.

### Study setting and health information system

According to the World Bank and the United Nations, the population residing in Lebanon reached 5,489,739 million in 2022 including 1,216,278 million Lebanese who were in precarious conditions and in need, on top of 1.5 million Syrian refugees [5,6]. Between 2019 and 2021, Lebanon’s gross domestic product (GDP) per capita significantly declined, leading to a reclassification of the country from an upper-middle-income country to a lower-middle-income country. The Ministry of Public Health (MOPH) used to cover about 53% of the population, it also supports a national network of PHCCs spread throughout Lebanon. These centers offer consultations with medical specialists at reduced costs, as well as medications and vaccines. Recently, a significant increase has been observed in the number of people turning to PHCCs for free medical care.

PHCCs in Lebanon operate using an electronic medical management software called Phenix, directly linked to the MOPH. This system aims to ensure controlled and supervised distribution of medications. For Hôtel Dieu de France PHCC, the adoption of this system began in 2022, which required manually collecting data for this study from paper medical records. At the Saint Antoine PHCC, the Phenix system has been in place for several years. Patient selection was based on the ICD10-I10 code (essential hypertension code) for the years 2019–2021. The selected records were then manually reviewed to eliminate any patients meeting an exclusion criterion. The Makhzoumi PHCC had its own system, similar to Phenix, with patient records also computerized. All records registered with the ICD10-I10 code for the years 2019–2020 were selected. Patients meeting exclusion criteria were excluded by reviewing the history and summary notes in their electronic medical record software.

### Eligibility criteria

This study included all patients aged 18 years and older who presented for the first time with a diagnosis of hypertension or were diagnosed with hypertension within a period of three months and had not yet started treatment.

Patients who were seen only once and did not have follow-up visits after the initial hypertension consultation, as well as those with chronic kidney disease stages 3–5, diabetes, or a history of cardiovascular events such as myocardial infarction, stroke, bypass surgery, or coronary stenting, were excluded.

### Sample size

It is estimated that the incidence of hypertension varies between 3% and 18% globally. Considering a 95% confidence interval with a 5% margin of error, and the population size of 5 million Lebanese, the sample size should be between 45 and 227 to be representative of patients with newly diagnosed hypertension.

### Data collection

Data were accessed between 1st June 2023 and 10th January 2024.

Data collected from patients’ medical records at baseline included demographics (age, sex, current smoking status), and clinical parameters such as systolic blood pressure (SBP) and diastolic blood pressure (DBP). Additionally, baseline paraclinical tests, if performed, were recorded based on WHO recommendations. These tests comprised eight items: serum sodium, serum potassium, serum creatinine, urine dipstick, glycated hemoglobin (HbA1c), fasting glucose, lipid profile, and ECG.

During the follow-up, we collected the incident comorbidities such as diabetes, chronic kidney disease and hypercholesterolemia. We collected SBP and DBP levels at 6 months, one year, and the last visit (the last visit defined as any visit that occurred beyond 12 months after the first consultation).

### Definitions

#### Hypertension.

Hypertension was defined as a blood pressure ≥ 140/90 mmHg.

#### Paraclinical tests.

Complete testing was defined as having the 8 tests performed. Partial testing was considered as getting 1–7 out of 8 tests. If a particular test was performed multiple times for the same individual during the follow-up and treatment period, only the first value was included in the data collection.

#### Abnormal laboratory tests.

Chronic kidney disease (stages 3–5) was defined as an estimated glomerular filtration rate (eGFR) < 60 mL/min/1.73 m² based on the CKD-EPI equation, LDL Cholesterol was considered elevated if higher than 100 mg/dL (>2.586 mmoL/L), and hypokalemia was defined as below 3.5 mmol/L. Diabetes was defined as an HbA1c above 6.5% or fasting blood glucose above 125 mg/dL (>6.938 mmoL/L).

### Statistical analysis

Statistical analysis was performed using the Statistical Package for the Social Sciences (SPSS), version 24.0. Continuous variables were presented as means and standard deviations (SD) if normally distributed, and as medians and interquartile ranges (IQR) if not normally distributed. Normality assumption was assessed using the Shapiro-Wilk test, the Kolmogorov-Smirnov test, and the histogram curve. Categorical variables were reported as numbers and percentages. The independent t-test and the Mann-Whitney U test were used to compare between two groups’ means and medians respectively. ANOVA was used to compare the three groups of testing (complete, partial and no testing) when the variable followed a normal distribution, and the Kruskal-Wallis test was used when the variables were not normally distributed. Bonferroni post-hoc analysis was performed to explore differences between sub-groups if ANOVA showed a significant difference. The Chi-square test was used to compare categorical variables. Subanalyses were performed to assess the difference between men and women and smokers and non-smokers. A *P* value < 0.05 was considered statistically significant.

### Ethical considerations

This study was approved by the ethics committee of Saint Joseph University (Tfem/2023/47) and was conducted in accordance with the Declarations of Helsinki of 1975. The ethics committee waived the requirement for informed consent.

## Results

### Inclusion of patients

In one of the three centers, all medical records were manually reviewed due to the absence of ICD10 classification. Over a 5-year period, 1648 patients were seen in this center, of whom 285 had the diagnosis of hypertension. Among these 285 patients, 148 with a prior history of hypertension were excluded, leaving 137 patients with new-onset hypertension. After excluding 101 patients with a history of cardiovascular or kidney disease or those lost to follow-up, 36 patients were included in the study. Based on data from this center, the incidence of hypertension was estimated at 83 per 1000 people.

In the other two centers, using the ICD10 code, we identified 336 patients with the diagnosis of hypertension. Out of these 336 patients, 174 were excluded due to a prior history of hypertension, leaving 162 patients with new-onset hypertension. Out of these 162 patients, 71 were included in our study after exclusion of 91 patients with a history of cardiovascular or kidney disease or loss to follow-up.

**Fig 1:**
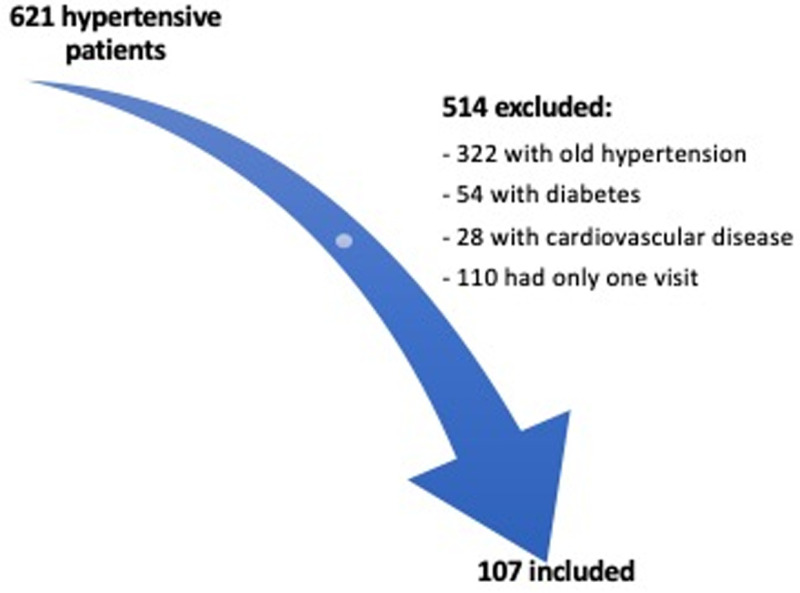
Flowchart of inclusion of patients.

The inclusion of patients is illustrated in Fig 1 and S1 File.

### General characteristics

The mean age of included patients was 54.8 ± 12.7 years, with 58.9% females (Table 1). The majority of patients (81.3%) were aged between 30 and 65 years old. 51.4% of patients were smokers. The mean SBP and DBP measured at the first visit (at the time of diagnosis) were respectively 153.1 ± 17.9 mmHg and 93.5 ± 10.4 mmHg.

**Table 1:** General characteristics of patients.

	Total sample N = 107	Full baseline testing n = 48	Partial testing n = 52	No testing n = 7	*P*
Number of tests, Median (IQR)	8 (8, 8)	8 (8, 8)	7 (7,7)	0	<0.001
Age in years Mean ±SD	54.8 ± 12.7	55.2 ± 11.6	54.4 ± 13.6	55.1 ± 15.6	0.954
Sex M/F, n(%)	47/63, (41.1/58.9)	28/20 (58.3/41.7)	15/37 (28.8/71.2)	1/6 (14.3/85.7)	0.004
Smoking, n(%)	55 (51.4)	31 (64.6)	22 (42.3)	2 (28.6)	0.038
Serum sodium, mmoL/L Median (IQR)	139 (138, 140)	140 (138, 141)	139 (137, 140)	NA	0.363
Serum potassium, mmoL/L Min-Max	4.5 ± 0.4 2.7-5.5	4.4 ± 0.3	4.5 ± 0.5	NA	0.511
Serum creatinine, mg/dL Median (IQR)	0.8 (0.7, 0.9)	0.8 (0.6, 0.8)	0.8 (0.7, 0.9)	NA	0.225
Glucose, mg/dL Median (IQR)	100 (92, 111)	99 (93, 115)	100 (90, 107)	NA	0.488
HbA1c, % Median (IQR)	5.6 (5.2, 5.9)	5.6 (5.2, 5.9)	5.5 (5.1, 5.7)	NA	0.120
LDL Cholesterol, mg/dL Mean ±SD	115.5 ± 42.3	109.3 ± 44.3	123.4 ± 38.9	NA	0.127
Total Cholesterol, mg/dL Mean ±SD	202.9 ± 43.2	196.3 ± 47.5	210.5 ± 36.9	NA	0.122
Urinalysis, n(%)	71 (66.4)	48 (100)	23 (44.2)	NA	<0.001
ECG, n(%)	85 (79.4)	48 (100)	37 (71.2)	NA	<0.001

Note. Variables with normal distribution were reported as mean ±standard deviation (SD) and those not normally distributed were reported as median and interquartile range (IQR).

One-way ANOVA test was used to compare the three groups when the variable followed a normal distribution, and Kruskal-Wallis test was used when variables were not normally distributed. Chi-Square test was used to compare categorical variables. The independent t-test and the Mann-Whitney U test were used to compare between two groups’ means and medians respectively. NA not applicable. IQR includes the 25th and 75th percentile.

### Number of tests performed at initial assessment

Among the 107 included patients, 48 underwent a complete initial assessment, while 52 had a partial assessment, and 7 patients did not undergo any tests. Among those who underwent a partial assessment, 20 patients had 7 tests, 11 had 6 tests, 5 had 5 tests, 5 had 4 tests, 5 had 3 tests, 2 had 2 tests and 4 had one test.

The percentage of patients who underwent the tests is as follows: 81% for serum creatinine, 80% for LDL cholesterol, 79.4% for serum sodium and potassium, 79.4% for HbA1c or glucose, 79.4% for ECG and 66.4% for urine dipstick.

### Abnormalities detected after laboratory testing

**Fig 2:**
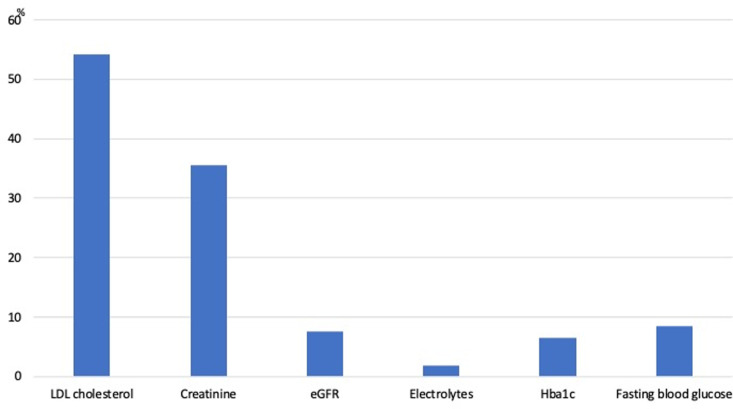
Percentage of abnormalities identified in the baseline laboratory assessment.

In our sample, 8.4% had fasting glucose levels exceeding 125 mg/dL, 7.5% had HbA1c levels above 6.5%, and 1.9% had serum potassium levels below 3.5 mmol/L (Fig 2). Additionally, 54.2% had LDL cholesterol levels above 100 mg/dL, 35.5% had serum creatinine levels above 0.8 mg/dL, and 7.5% had an eGFR below 60 mL/min/1.73 m².

### Blood pressure control

There was a significant improvement in SBP control after 12 months of diagnosis among individuals who underwent complete testing compared to those who underwent partial testing, with mean values of 129.9 ± 13.6 mmHg and 142.8 ± 18.9 mmHg respectively (*P* = 0.003) (Table 2). Bonferroni post-hoc analysis showed a significant difference between the complete testing and no testing groups (*P* = 0.003) and between the partial testing and the no testing groups (*P* = 0.003).

**Table 2:** Comparison of blood pressure levels across different categories of baseline testing.

	Total sample N = 107	Full baseline testing n = 48	Partial testing n = 52	No testing n = 7	*P*
Baseline SBP, mmHg Mean ±SD Median (IQR)	153.1 ± 17.9 150 (140, 160)	152.4 ± 16.4 149 (140, 160)	154.3 ± 19.6 150 (140, 160)	149.6 ± 16.7 150 (137, 160)	0.753
Baseline DBP, mmHg Mean ±SD	93.5 ± 10.4	92.9 ± 10.3	94.5 ± 10.6	90.3 ± 12.3	0.544
SBP at 6 months, mmHg Mean ±SD	132.6 ± 12.7	131.8 ± 12.7	134 ± 12.6	129 ± 14.7	0.674
DBP at 6 months, mmHg Mean ±SD	81.5 ± 9.9	81.3 ± 10.4	81.3 ± 9.7	84.8 ± 10	0.510
SBP at 12 months, mmHg Mean ±SD Median (IQR)	135.8 ± 17 135 (125,140)	129.9 ± 13.6 130 (120, 140)	142.8 ± 18.9 140 (130, 150)	132.3 ± 11.6 130 (120, 140)	0.003
DBP at 12 months, mmHg Mean ±SD	84.1 ± 11	81.8 ± 9.8	86.7 ± 12.2	83.7 ± 9.9	0.163
SBP at last visit, mmHg Mean ±SD	134.5 ± 16.2	132.5 ± 17.4	136.1 ± 16.2	135.7 ± 5.5	0.549
DBP at last visit, mmHg Mean ±SD	81.6 ± 12.1	78.6 ± 11.3	83.6 ± 12.3	86 ± 12.1	0.077
BP controlled at last visit, n(%)	66 (61.7)	30 (62.5)	30 (57.7)	6 (85.7)	0.445

Note. Variables with normal distribution were reported as mean ±standard deviation (SD) and those not normally distributed were reported as median and interquartile range (IQR).

One-way ANOVA test was used to compare the three groups when the variable followed a normal distribution, and Kruskal-Wallis test was used when variables were not normally distributed. The independent t-test and the Mann-Whitney U test were used to compare between two groups’ means and medians respectively. Chi-Square test was used to compare categorical variables. IQR includes the 25th and 75th percentile.

**Fig 3:**
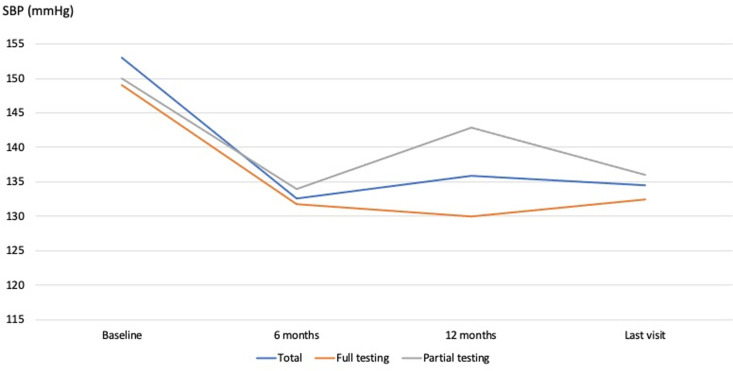
Systolic blood pressure control at 6 months, 12 months and last visit.

No significant difference was observed for DBP at 12 months (*P* = 0.163). However, there was a trend towards significance for DBP measurements at the last visit (*P* = 0.077). The mean DBP values were 78.6 ± 11.4 mmHg, 83.6 ± 12.3 mmHg, and 86 ± 12.1 mmHg in the groups with complete, partial, and no initial testing, respectively (Fig 3).

Regarding overall blood pressure control at the last visit, no difference was observed among the three groups. In our sample, blood pressure control was achieved in 61.7% of patients.

Among patients with a decreased eGFR below 60 mL/min/1.73 m², there was a significant difference in blood pressure control; 75% did not have their blood pressure controlled (*P* = 0.014).

When comparing men and women, the blood pressure control was not different at the last visit between men and women (65.9% in men versus 58.7% in women *P* = 0.680).

### Subanalyses

#### Correlation between baseline testing and sex.

**Fig 4:**
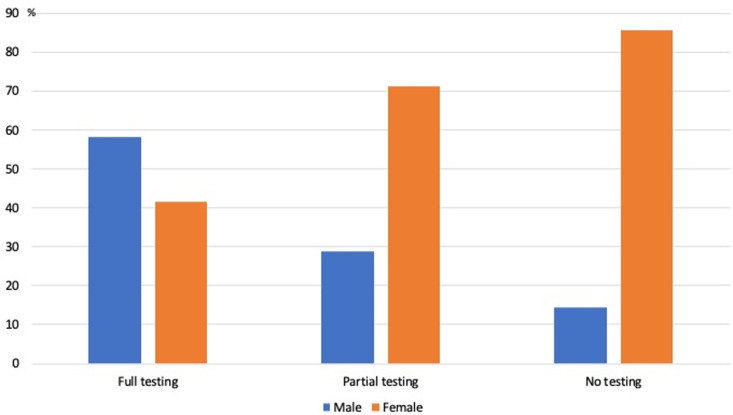
Differences in testing between men and women.

A statistically significant difference was observed in the number of tests performed between men and women (*P* = 0.004). Men underwent more testing than women, with 58.3% of men in the group receiving a complete initial assessment compared to 41.7% of women. In the partial testing group and no-testing group, the percentage of women reached 71.2% and 85.7%, respectively, compared to 28.8% and 14.3% of men (Fig 4).

#### Correlation between baseline testing and smoking status.

**Fig 5:**
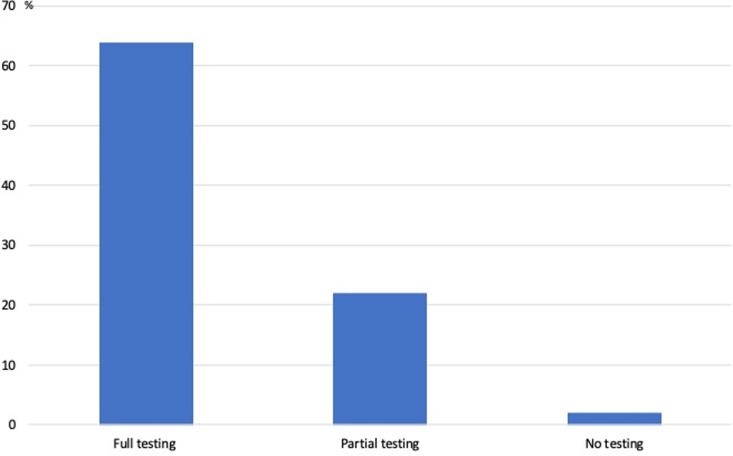
Percentage of testing based on smoking status.

Smokers underwent more tests, with 64% of smokers in the group undergoing a complete assessment, compared to proportions of 22% and 2% in the partial testing and no-testing groups, respectively (*P* = 0.038) (Fig 5).

## Discussion

This study revealed that the incidence of hypertension is 8.3% in primary care settings. Approximately 25% of these patients with new-onset hypertension have isolated hypertension, without any other known comorbidities. The average age of these patients was 55 years, figures comparable to those observed in Latin America and the Middle East, with respective averages of 52 years and 49 years [7,8]. While hypertension is often associated with other comorbidities, it can also manifest alone without other medical conditions. It is crucial to assess this category of hypertensive patients and analyze the importance of comprehensive evaluation in controlling blood pressure levels and assessing associated hidden comorbidities.

Our study showed that baseline paraclinical assessment unmasked chronic kidney disease in 7.5% of patients with new-onset hypertension, diabetes in another 7.5%, dyslipidemia in 54.2%, and significant electrolyte abnormalities (serum potassium <3.5 mmoL/L) in less than 2%. A study conducted in Alberta, Canada, in 2020 highlighted various anomalies among hypertensive patients, including 10% with renal abnormalities, 0.14% with electrolyte abnormalities, 20% with elevated LDL levels > 3.5 mmoL/L (>135 mg/dL), and 50% with elevated HbA1c and fasting blood glucose levels [9]. Furthermore, a study carried out in Portugal in 2019 examined the prevalence of cardiovascular risk factors and other comorbidities among hypertensive patients attending primary health care centers. Hypercholesterolemia was found to be the most common concurrent cardiovascular risk factor, affecting 82.1% of participants [10]. Although the study in Alberta, Canada, used serum creatinine as an indicator of kidney function, the prevalence of kidney abnormalities in their results remained close to that observed in our study, with respective percentages of 10% and 7.5%. Regarding LDL levels, a difference was noted, with only 20% of LDL abnormalities in Alberta compared to 54.2% in our study and 82.1% in Portugal but this is related to different cut-offs used [10].

Interestingly, the less frequently performed tests in our study were electrocardiogram (ECG) and urinalysis. This significant disparity was also noted in a study on hypertension management in primary care across eight regions in Sweden. Results showed that the rate of ECGs performed during the initial visit was 79%, while urinalysis was 47%, percentages lower than those recorded for creatinine (100%), potassium (96%), lipid profile (95%), and blood glucose (92%) [11]. This could be attributable to the absence of ECG machines in the centers. A study conducted in Jordan assessed the capabilities of 23 primary care centers for managing hypertension. ECG machines were available in all centers except two [7]. In Lebanese PHCCs, testing may have been influenced by both physician and patient preferences. The lack of awareness about guidelines, the absence of local guidelines, and insufficient education and ongoing training might also have contributed to the observed testing pattern in Lebanon. ECG machines are generally available in PHCCs, and we assume that ECG testing was not performed in all cases due to medical decisions or time constraints during the visit. Regarding the urine dipstick, reasons for not performing it could be related to hygiene concerns or patient refusal.

One of the objectives of our study was to examine the impact of initial diagnostic assessments for hypertension on blood pressure control. It was found that patients who underwent a comprehensive baseline assessment had better systolic blood pressure control at 12 months post-diagnosis, with an average of 129.9 ± 13.6 mmHg compared to 142.8 ± 18.9 mmHg in the group that received partial initial assessments. This difference may be due to screening for comorbidities commonly associated with hypertension, such as newly diagnosed diabetes or chronic kidney disease, or simply dyslipidemia. This leads to better blood pressure management by targeting various aspects of the patient’s cardiovascular profile. In addition to detecting coexisting comorbidities, the laboratory abnormalities identified during initial testing could influence the choice of one antihypertensive medication over another. For instance, angiotensin II receptor antagonists and angiotensin-converting enzyme inhibitors may be more appropriate for use in chronic kidney disease, diabetes, and atrial fibrillation. Diuretics, especially thiazides, can affect serum and urinary sodium concentrations, while angiotensin-converting enzyme inhibitors and angiotensin receptor blockers can influence blood biomarkers of the renin-angiotensin-aldosterone system (RAAS), such as serum potassium levels and creatinine [12]. It is noteworthy that no significant difference in blood pressure control was noted at the last visit in our study. One possible explanation is that most patients ultimately underwent comprehensive testing long after their initial hypertension diagnosis, which may have contributed to improved blood pressure control across all groups by the final visit. Additionally, treatment adherence may have changed over time. However, evaluating adherence in a retrospective study is challenging, and interventional studies are needed to address this issue.

Although the percentage of electrolyte disorders, particularly hypokalemia, is low in our study (1.9%) and in the study conducted in Alberta (0.14%), primary aldosteronism (PA), the most common endocrine disorder in secondary hypertension, actually represents a significant proportion of hypertensive individuals in absolute terms, despite its small relative incidence compared to overall hypertension. However, it affects a younger population than our sample.

In terms of blood pressure control rates in our study, a high rate of 61.7% was observed at the last visit, a significantly high percentage comparable to control levels observed in developed countries (60–70%) [10]. In contrast, these rates are much lower in other regions of the world, with figures of 37.6%, 19%, 3.5%, 16.4%, and 12% in the Middle East, Accra, Cameroon, Latin America and China respectively [7,12–15]. In these Middle Eastern countries, and in Accra and Cameroon, women showed better control of their blood pressure. In our sample, women had less blood pressure control at the last visit than men but without reaching statistical significance, probably because of our small sample size.

Interestingly, despite ongoing economic challenges in the country, most patients benefited from an initial assessment. Among the 107 patients included in our sample, 44.8% underwent a comprehensive assessment, while 48.5% had a partial assessment, leaving only 6.7% of patients without any assessment. These figures are comparable to a study conducted in Alberta, Canada in 2020, where 42.3% of participants completed all four recommended laboratory tests for a considered comprehensive assessment, with 32% undergoing a partial assessment and 25% without any assessment. Access to healthcare, even during economic crises, may be attributed to the significant contributions of numerous NGOs that address the needs of the population, particularly those living in more precarious conditions. Indeed, the costs of blood tests were covered by a third-party payer in two out of the three centers examined. Future research is essential to evaluate the cost barriers to implementing routine baseline testing in patients with new-onset hypertension in lower-resource settings.

Surprisingly, there was a higher prevalence of testing among men compared to women, with a statistically significant difference between the two sexes. This may reflect inequality in access to healthcare. According to the WHO, gender inequality and discrimination present substantial threats to women’s health and well-being, creating significant barriers to accessing essential health information and services. These barriers include restrictions on mobility and transportation, opportunities for education and employment, discriminatory attitudes among healthcare providers, exclusive focus on women’s reproductive roles, and a general lack of training and awareness in healthcare systems regarding the specific needs and challenges faced by women. Addressing these complex issues is essential to dismantling barriers and ensuring equitable access to healthcare services, enabling women and girls to achieve the highest possible level of health [16,17]. A Canadian study supports this hypothesis, revealing a prevalence of 12.0% for perceived unmet healthcare needs, with a significantly higher percentage among women compared to men (13.7% vs. 10.1%; P < 0.001) [18]. In our Lebanese sample, the reasons behind testing disparity among sexes are not entirely clear and may stem from behavioral or economic factors.

Finally, screening was more frequent among smokers. These two findings could be related to the fact that men smoke more than women. A study published in Guatemala in 2022 highlights that the prevalence of smoking among men was twenty times higher than among women (20.5% vs. 1.2%, respectively) [19]. Similarly, a 2022 study comparing metabolic, behavioral, and psychosocial risk factors, as well as cardiovascular diseases between women and men in 21 high-income, middle-income, and low-income countries, confirmed this point by indicating that overall, the population attributable fractions (PAFs) for behavioral and psychosocial risk factors were higher in men (15.7%) than in women (8.4%), mainly due to the greater contribution of smoking among men (10.7% vs. 1.3% among women) [20].

## Strengths and limitations

The strength of this study lies in its multicenter design and focus on patients without comorbidities, a group often underepresented in research. By excluding patients with comorbidities such as diabetes, cardiovascular disease, or prior hypertension, the study isolates the effects of essential hypertension, enhancing accuracy and providing clearer insights. This approach supports clinical decision-making by offering targeted data to guide personalized treatment strategies, ultimately improving patient care and outcomes.

Nevertheless, there are areas for improvement, such as expanding the scope of the study to include centers outside the capital city, incorporating rural centers into the analysis, standardizing blood pressure protocols and ensuring uniform follow-up times. Additionally, the financial support provided to two of the three centers by multiple NGOs may have influenced the ease of conducting assessments, particularly in a country where healthcare largely depends on private insurance. Another limitation is the study’s retrospective design which may introduce biases. Potential confounders, such as socioeconomic status and educational level, could also have impacted the findings and warrant consideration in future research. We finally acknowledge two limitations: the no-testing group was very small and the timing of the last blood pressure measurement (above 12 months) was different across patients and this heterogeneity may have affected the non-significant results.

## Conclusion

Our study highlights the critical role of early baseline laboratory testing in newly diagnosed hypertensive patients and supports routine baseline laboratory testing for all these hypertensive patients. This assessment not only facilitates improved blood pressure control at one year but also identifies significant comorbidities including diabetes, hypercholesterolemia and chronic kidney disease. By uncovering these conditions early, this proactive approach can guide the selection of antihypertensive therapies and optimize personalized patient management. Furthermore, the findings from this retrospective study underscore the need for validation in larger cohorts, as well as cost-effectiveness analyses, with extended follow-up periods to evaluate long-term outcomes, particularly survival benefits.

## Supporting information

S1 File Distribution of patients. (PDF)
