# Supplementary material for: The impact of baseline laboratory tests on the management of new-onset hypertension in primary care: A pilot study
Source: PLoS One. 2025 May 29;20(5):e0324743. doi: 10.1371/journal.pone.0324743 (PMC12121813; doi:10.1371/journal.pone.0324743)
Supplement: S1 File — (PDF) [file pone.0324743.s001.pdf]

S1 File. Distribution of patients

At Hôtel Dieu de France, we reviewed a total of 1648 records, including all diagnoses. Among these, 285 patients were diagnosed with hypertension, of which 36 had essential hypertension without any other cardiovascular risk factors, constituting the sample for this study. Conversely, 249 patients were excluded: 148 were already hypertensive before this visit, 23 were diabetic, 15 had cardiovascular histories, and 63 had only one visit, resulting in loss to follow-up.

At the Saint Antoine - Jdeideh dispensary, we reviewed a total of 177 records of individuals registered in the Phenix system as having essential hypertension, ICD10 code I10. Among these records, 53 were included. Of the 124 excluded records, 77 were already hypertensive, 19 were diabetic, 6 had cardiovascular histories, and 22 had only one visit, resulting in loss to follow-up.

At the Makhzoumi center, we reviewed a total of 159 records of individuals registered in their software as having essential hypertension, ICD10 code I10. Among these records, 18 were included. Of the 141 excluded records, 97 were already hypertensive, 12 were diabetic, 7 had cardiovascular histories, and 25 had only one visit, resulting in loss to follow-up.
